# Sodium Glucose Cotransporter 2 (SGLT2) Inhibitor Ameliorate Metabolic Disorder and Obesity Induced Cardiomyocyte Injury and Mitochondrial Remodeling

**DOI:** 10.3390/ijms24076842

**Published:** 2023-04-06

**Authors:** Shih-Jie Jhuo, Yi-Hsiung Lin, I-Hsin Liu, Tsung-Hsien Lin, Bin-Nan Wu, Kun-Tai Lee, Wen-Ter Lai

**Affiliations:** 1 Graduate Institute of Clinical Medicine, College of Medicine, Kaohsiung Medical University, Kaohsiung 807, Taiwan; 2 Division of Cardiology, Department of Internal Medicine, Kaohsiung Medical University Hospital, Kaohsiung 807, Taiwan; 3 Department of Internal Medicine, Faculty of Medicine, College of Medicine, Kaohsiung Medical University, Kaohsiung 807, Taiwan; 4 Lipid Science and Aging Research Center, Kaohsiung Medical University, Kaohsiung 807, Taiwan; 5 Center for Lipid Biosciences, Kaohsiung Medical University Hospital, Kaohsiung 807, Taiwan; 6 Department of Pharmacology, Graduate Institute of Medicine, College of Medicine, Drug Development and Value Creation Research Center, Kaohsiung Medical University, Kaohsiung 807, Taiwan; 7 Department of Medical Research, Kaohsiung Medical University Hospital, Kaohsiung 807, Taiwan

**Keywords:** SGLT2 inhibitor, empagliflozin, epicardial fat, mitochondria, oxidative stress, calcium overload

## Abstract

Sodium-glucose transporter 2 inhibitors (SGLT2is) exert significant cardiovascular and heart failure benefits in type 2 diabetes mellitus (DM) patients and can help reduce cardiac arrhythmia incidence in clinical practice. However, its effect on regulating cardiomyocyte mitochondria remain unclear. To evaluate its effect on myocardial mitochondria, C57BL/6J mice were divided into four groups, including: (1) control, (2) high fat diet (HFD)-induced metabolic disorder and obesity (MDO), (3) MDO with empagliflozin (EMPA) treatment, and (4) MDO with glibenclamide (GLI) treatment. All mice were sacrificed after 16 weeks of feeding and the epicardial fat secretome was collected. H9c2 cells were treated with the different secretomes for 18 h. ROS production, Ca^2+^ distribution, and associated proteins expression in mitochondria were investigated to reveal the underlying mechanisms of SGLT2is on cardiomyocytes. We found that lipotoxicity, mitochondrial ROS production, mitochondrial Ca^2+^ overload, and the levels of the associated protein, SOD1, were significantly lower in the EMPA group than in the MDO group, accompanied with increased ATP production in the EMPA-treated group. The expression of mfn2, SIRT1, and SERCA were also found to be lower after EMPA-secretome treatment. EMPA-induced epicardial fat secretome in mice preserved a better cardiomyocyte mitochondrial biogenesis function than the MDO group. In addition to reducing ROS production in mitochondria, it also ameliorated mitochondrial Ca^2+^ overload caused by MDO-secretome. These findings provide evidence and potential mechanisms for the benefit of SGLT2i in heart failure and arrhythmias.

## 1. Introduction

Metabolic syndrome (MS) is a well known risk factor for cardiac arrhythmia and the remodeling of cardiac substrates in patients with metabolic-disorder-induced obesity may be a cause [1]. Metabolic disorder and obesity (MDO) increases the amount of visceral fat, which is associated with the occurrence of cardiac arrhythmia [2]. Previous studies have suggested that adipose tissues provide several cytokines and proinflammatory mediators that modulate the genesis of abnormal cardiac rhythms [3,4]. Our previous study also demonstrated that secretomes from epicardial fat can modulate the ion currents of cardiomyocytes, which may result in arrhythmogenesis [5,6].

Sodium-glucose transporter 2 inhibitors (SGLT2is) have emerged as next-generation glucose-lowering agents in recent years [7]. Beyond exerting glucose-lowering effects, SGLT2is also significantly decreases cardiovascular mortality and heart failure hospitalizations for type 2 diabetes mellitus (DM) patients with high cardiovascular risk [8]. In the DAPA-HF trial, patients with reduced ejection fraction had a significantly reduced incidence of cardiovascular death and hospitalization after SGLT2i therapy [9]. As we have previously reported, SGLT2i therapy also leads to significantly reduced incidences of atrial fibrillation and total cardiac arrhythmia in clinical practice [10]. Notably, previous studies have revealed that SGLT2is do not prolong the QT interval on electrocardiography, alleviate atrial remodeling, or improve mitochondrial function in diabetic rats [11,12]. Several possible mechanisms have been discussed, but none have been fully investigated or deemed satisfactory.

The production of reactive oxygen species (ROS) causes sustained oxidative stress (OS) in cardiomyocytes, which is associated with cardiovascular disease and cardiac arrhythmia [13]. ROS in mitochondria are generated by the single-electron reduction of molecular oxygen (O_2_) during oxidative phosphorylation [14]. ROS levels are significantly elevated when the cardiac workload and OS in cardiomyocytes are increased during heart failure. ROS are also significantly associated with cardiac fibrosis [15], which might have arrhythmogenic effects. The association of ROS with mitochondria has been extensively discussed. Excessive ROS production causes instability of ion currents, which might result in cardiac arrhythmia [16,17,18]. Mitochondrial homeostasis of calcium ions (Ca^2+^) and ROS is crucial in preventing cardiac arrhythmia. Recent studies have revealed that suppression of mitochondrial ROS production after SGLT2i therapy [19,20] might have a protective effect on cardiomyocytes. However, the exact mechanisms remain unclear. In this study, we attempted to elucidate the effect of SGLT2i empagliflozin (EMPA) on ameliorating the adverse effects of the MDO-epicardial fat secretome on cardiomyocytes. This study could provide basic experimental data regarding the effect of EMPA in regulating cardiomyocyte H9c2 cell mitochondrial function, and evidence for the benefits of EMPA in the prevention and treatment of associated cardiovascular diseases, such as heart failure and arrhythmia.

## 2. Results

### 2.1. The Epicardial Fat Secretome Induces Cardiotoxicity and Mitochondrial Dysfunction

**Figure 1:**
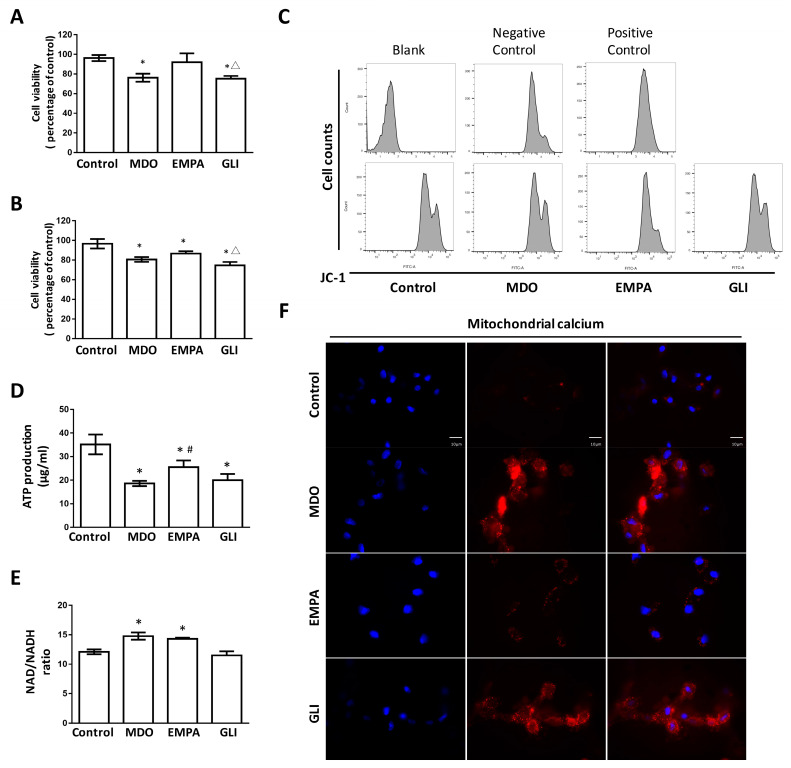
Effects of the pericardial fat secretome of different groups of metabolic-syndrome-associated mice on cardiomyocyte H9c2. Secretome of pericardial fat on H9c2 cell viability inhibition at (**A**) 48 h and (**B**) 72 h. (**C**) The effect of secretome on mitochondrial membrane potential alteration of H9c2. 50 ug/mL CCCP was used as positive control. (**D**) ATP production capacity and (**E**) NAD^+^/NADH ratio were determined as the evaluation of H9c2 mitochondrial function after treatment with different groups’ pericardial fat secretome. (**F**) Rhod-2 AM staining for intra-mitochondria Ca^2+^ distribution (Magnification: 400×). In vitro study, *n* = 3 for each group, * *p* < 0.05 compared with control group, # *p* < 0.05 compared with MDO group, Δ *p* < 0.05 compared with EMPA group.

Fat-/lipid-derived lipotoxicity is known to affect many organs and tissues. In our study, H9c2 cells were used to investigate the effect of the epicardial fat secretome on the growth of ventricular cardiomyocytes. As shown in Figure 1A, we found that MDO group-derived fat exhibited lipotoxicity, effectively inhibiting the growth of ventricular cardiomyocyte H9c2 cells. In cells cultured for 48 h, the secretome extracted from the MDO group inhibited 20% cell growth more than the secretome extracted from the control group. In contrast, the secretome from the EMPA group exhibited lower cardiac lipotoxicity. The secretome from the glibenclamide (GLI) group had the same growth-inhibitory effect as the MDO group and also inhibited the growth of H9c2 cells by about 20%. However, although the EMPA group-derived secretome attenuated growth inhibition, after 72 h, growth inhibition was not significantly different between the EMPA secretome-treated cells and the MDO secretome-treated cells (Figure 1B). These results showed that 10 µg/mL EMPA elicited the best response under 48 h of secretome culture. Previous studies have indicated that SGLT2i therapy can affect gene expression related to mitochondrial function [21]; however, mitochondria in MDO fat-stressed ventricular cells have not yet been examined. Therefore, mitochondrial function was investigated with JC-1 staining. Figure 1C shows that the mitochondrial function of ventricular cardiomyocytes was significantly impaired in the MDO secretome-treated group compared with the normal group. The MDO secretome implicated 46% of the mitochondrial ATP production in cardiomyocyte H9c2 cells. (Figure 1D). Consistent with this implication, co-treatment with the EMPA secretome effectively attenuated the MDO-fat secretome-induced reduction in mitochondrial ATP production, preserving approximately 71% of the ATP production capacity compared with the control group. However, our assessment of mitochondrial NADH biosynthesis showed that EMPA did not significantly alter the NADH/NAD^+^ ratio that was increased by MDO epicardial fat in mitochondria (Figure 1E). The secretome of both the MDO group and the EMPA group enhanced NADH biosynthesis in H9c2 by approximately 25%. Considering our previous published findings that the fat secretome affects the Ca^2+^ currents of cardiomyocytes, the Ca^2+^ distribution in the mitochondria of H9c2 cells was further investigated. Staining with Rhod-2 AM and fluorescence microscopy revealed that MDO-derived fat induced considerable accumulation of Ca^2+^ in mitochondria, but the accumulation was significantly reduced in the EMPA group. GLI treatment also slightly but insignificantly reduced intra-mitochondrial Ca^2+^ deposition (Figure 1F). These results suggested that the MDO secretome caused mitochondria-related cardiotoxicity and increased intramitochondrial Ca^2+^ accumulation, which may have led to Ca^2+^ overload in cardiomyocytes and caused intracellular stress. However, the EMPA secretome effectively attenuated mitochondrial dysfunction and improved the survival rate of cardiomyocytes.

### 2.2. Mitochondrial Dysregulation Leads to Increased Intracellular ROS Production

**Figure 2:**
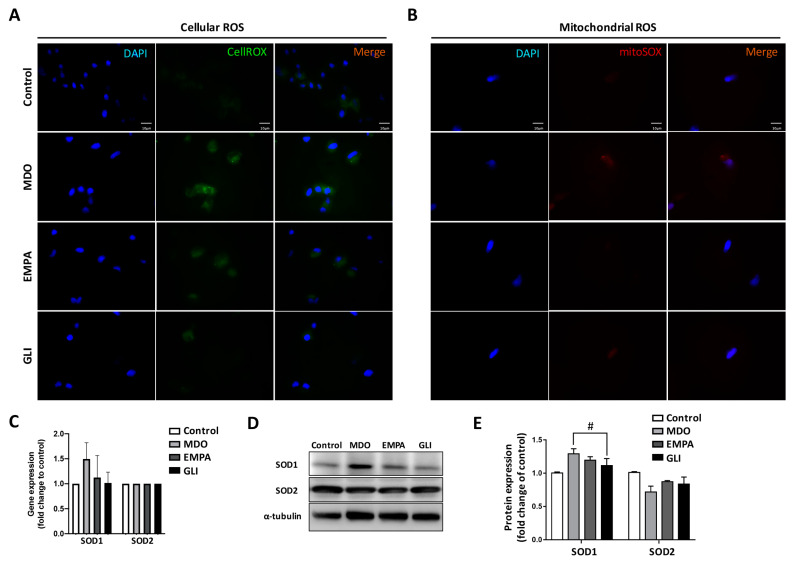
ROS production and antioxidant expression in response to oxidative stress in H9c2 cells. Effects of epicardial fat secretome on the production and accumulation of (**A**) cellular ROS and (**B**) intra-mitochondria ROS of H9c2 cells. (Magnification: 400×) (**C**) The OS-associated antioxidant SOD1 and SOD2 genes in H9c2 cells respond to the secretome. (**D**) Protein expression changes and (**E**) quantitative results of antioxidants SOD1 and SOD2 in H9c2 cells. In vitro study, *n* = 3 for each group, # *p* < 0.05 compared with MDO group.

We further investigated the mitochondrial dysregulation induced by secretomes from epicardial fat. Since mitochondrial dysfunction is highly associated with ROS production, the expression of intracellular ROS among the study groups was investigated. As shown in Figure 2A, CellROX-staining to investigate the intracellular ROS distribution revealed that ROS in H9c2 cells accumulated in the cytoplasm after MDO secretome treatment. The MDO secretome-induced production and accumulation of ROS in mitochondria was also investigated. Figure 2B shows that mitochondrial ROS levels also increased significantly after MDO secretome treatment, indicating that the MDO secretome increased ROS in both whole H9c2 cells and mitochondria. Co-treatment with the EMPA secretome effectively reduced accumulation of intracellular and intramitochondrial ROS, suggesting that SGLT2i reduced the intracellular OS caused by MDO fat and improved cardiomyocyte survival. We then examined the gene and protein expression of the OS-related regulatory factors, SOD1 and SOD2 (Figure 2C–E). The gene expression and protein expression of SOD1 in the MDO group increased significantly by 1.5-fold and 1.34-fold, respectively. However, compared with the control group, neither the gene nor the protein of SOD1 increased significantly in the EMPA group. There was no significant change in SOD2 regardless of group. These results indicated that the MDO secretome increased ROS production and the expression of SOD1 under OS, but these effects were attenuated by the EMPA and GLI secretomes.

### 2.3. Effects of the EMPA Secretome on Mitochondrial Function Regulators

**Figure 3:**
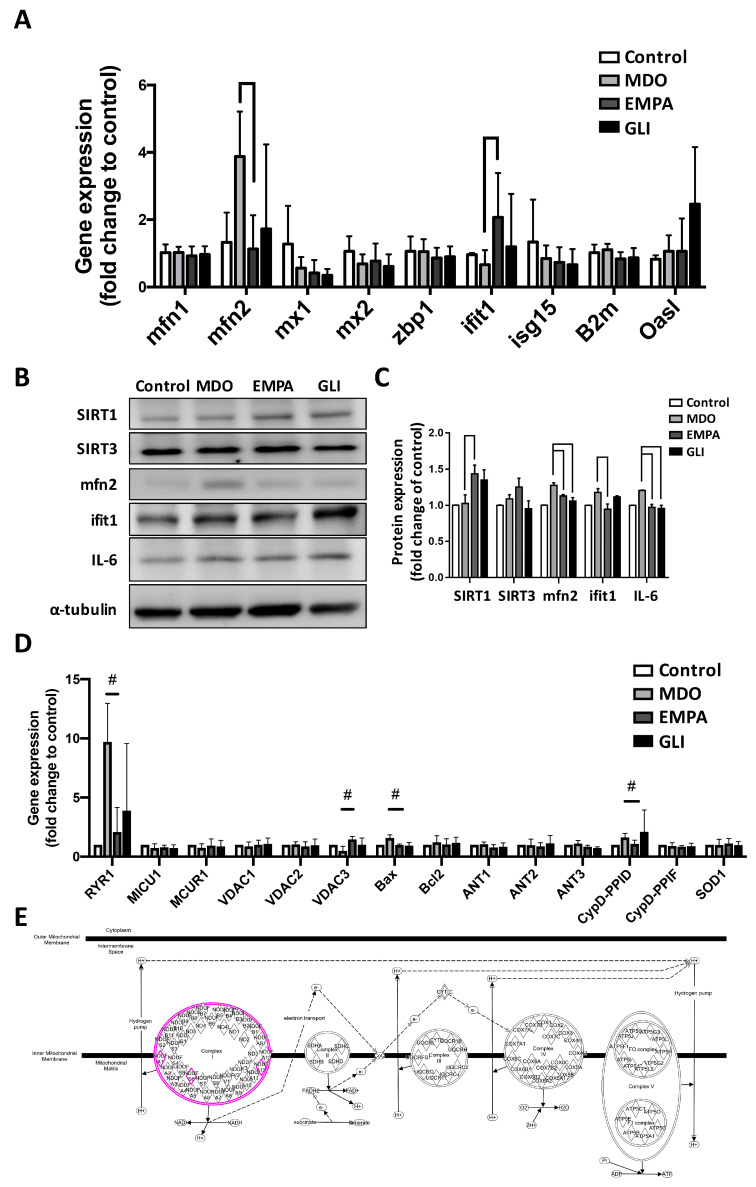
MDO- and EMPA-associated epicardial fat secretome in genes regulation correlated with mitochondrial function. (**A**) The expression alteration of regulatory genes related to mitochondrial fission and fusion of H9c2 treated by different epicardial fat secretomes. Western blot analysis of (**B**) protein expression and (**C**) the quantitative results of regulators involved in secretome-manipulated H9c2 mitochondrial function. (**D**) The impact of fat secretomes on the gene expression of Ca^2+^ channel regulators in mitochondria. # *p* < 0.05 compared with MDO group (**E**) KEGG pathway analysis of RNA-sequencing predictions’ potential gene network affected by the epicardial fat secretome. (In vitro study, *n* = 3 for each group).

The effects of the MDO secretome on the mitochondria in H9c2 cells were evaluated. Specifically, we investigated the associated regulators of mitochondrial fission and fusion after secretome treatment. According to qPCR analysis of mitochondrial-homeostasis-related genes, mfn2 and ifit-1 responded the most prominently to EMPA secretome treatment. We found that mfn2 was increased approximately 3.8-fold in the MDO group but was attenuated in the EMPA group to be similar to the control group. The same trend was also found in the GLI group; however, it was not statistically significant. In contrast, ifit-1 gene expression was not significantly different in the MDO group but had increased approximately 2-fold in the EMPA group. There was also no significant change in the GLI group (Figure 3A). The protein expression of mfn2 and ifit-1 were also studied. Similar to the results of gene expression, the mfn2 protein expression of the MDO group was enhanced by about 1.3 times, while that of the EMPA group was reduced to no difference from the control group. In addition, ifit-1 protein expression was increased in the MDO group, while it was slightly decreased in the EMPA group. Ifit-1 protein expression was also higher in the GLI group than in the control group. We also investigated SIRT1 expression in H9c2, and it was found to be unchanged after MDO secretome treatment. However, SIRT1 expression was significantly increased by 1.48-fold in the EMPA group than in the control and MDO group. Interestingly, the secretome did not alter SIRT3 protein expression in the H9c2 cells. (Figure 3B,C). Our previous study suggested that the Ca^2+^ current is affected by MDO fat, thus, genes related to the regulation of mitochondrial Ca^2+^ handling remodeling were also investigated in this study. As shown in Figure 3D, a panel of regulatory genes associated with the regulation of mitochondrial Ca^2+^ flow and channels, including RYR1 and VDAC family genes, was examined. We found that the RYR1, Bax, and PPID genes were highly expressed under MDO secretome stimulation by 9.8-fold, 1.6-fold, and 1.7-fold, respectively. However, after treatment with secretome in the EMPA group, all decreased to the levels close to that of the control group. VDAC3 gene expression decreased by about 60% in the MDO group but increased 1.2-fold in the EMPA group compared with the control group. RNA-seq prediction revealed additional underlying regulatory genes involved in the restoration of mitochondrial function after EMPA secretome treatment, including the mitochondrial complex genes I-NDUFA1, NDUFS6, and NDUFA12. These results showed that MDO-related secretomes caused Ca^2+^ handling remodeling, and the capability of the EMPA-related secretome to restore mitochondrial homeostasis and Ca^2+^ handling.

### 2.4. RNA-Seq Analysis of the Effects of the EMPA Secretome on H9c2 Cells

**Figure 4:**
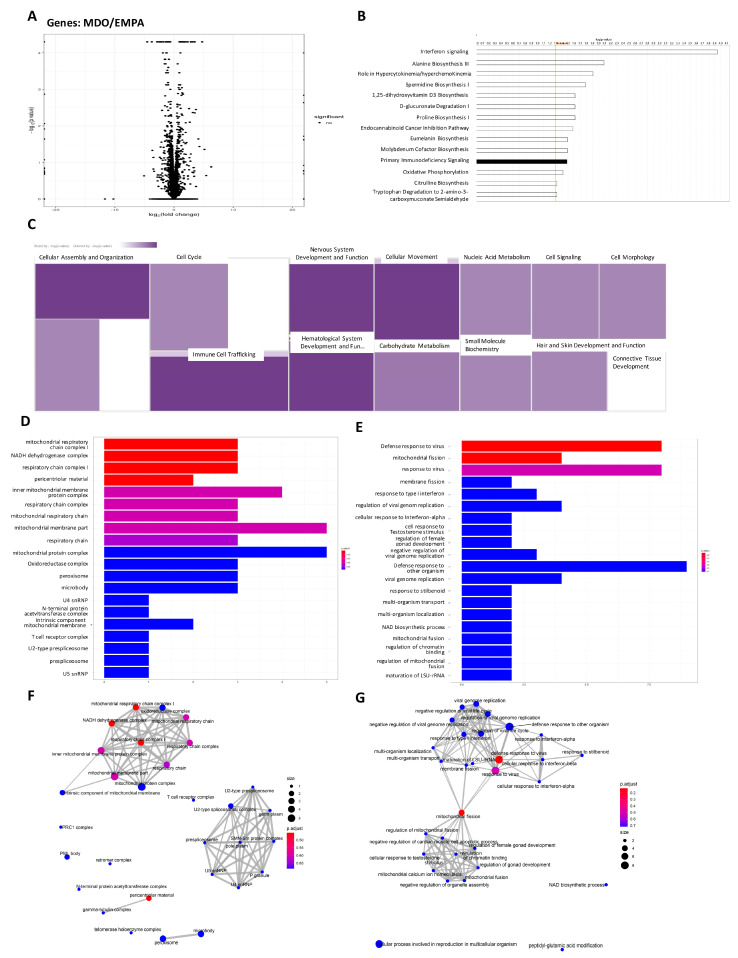
RNA-sequencing analysis of the impact of epicardial fat secretomes on H9c2 cellular process. (**A**) Volcano pots of differentially expressed genes (DEGs). (**B**) Canonical Pathway View and (**C**) Disease & Functions comparative analysis of DEGs’ participation. The analysis of DEGs in (**D**) cellular component (CC) and (**E**) biological process (BP) involved in cellular process were revealed. The gene regulatory network of (**F**) CC and (**G**) BP were shown by emap plot. (In vitro study, *n* = 3 for each group).

Having elucidated the effects of the EMPA secretome on H9c2 mitochondria, we still needed to perform a comprehensive analysis. We analyzed transcriptome modifications by using RNA-seq to clarify the underlying mechanism of the EMPA secretome in MDO stress. The volcano plot in Figure 4A shows the genes that were affected in the EMPA group. Among the total of 296 genes that were affected, 212 genes were upregulated in the EMPA group and 84 genes were downregulated. The DEGs were then subjected to canonical pathway analysis, and the most affected signal transduction pathway was the interferon signaling pathway. DEGs were also involved in oxidative phosphorylation, sirtuin signaling, xenobiotic metabolism, and mitochondrial dysfunction in H9c2 cells (Figure 4B). When we used the gene functions to analyze the correlations with cell functions, we found that the EMPA secretome enhanced most mechanisms of cell proliferation, such as cellular assembly and organization, cell cycling, cellular movement, and carbohydrate metabolism (Figure 4C). We next analyzed the effects of the EMPA secretome on the biological processes (BPs) and cellular components (CCs) of H9c2 cells. We found that genes altered by the EMPA secretome were associated with BPs and CCs related to mitochondrial regulation, such as the mitochondrial respiratory chain and complex I, the NADH dehydrogenase complex, and the mitochondrial membrane protein complex. They were also involved in cellular processes, such as mitochondrial fission, mitochondrial fusion, and the response to interferon signaling (Figure 4D–G). These results suggest that the EMPA secretome improves mitochondrial function by enhancing related signal transduction pathways in response to MDO stress, which might provide cardiomyocyte protection.

### 2.5. Verification of the Effects of the EMPA Secretome on the Myocardial Ventricle in the MDO Mouse Model

**Figure 5:**
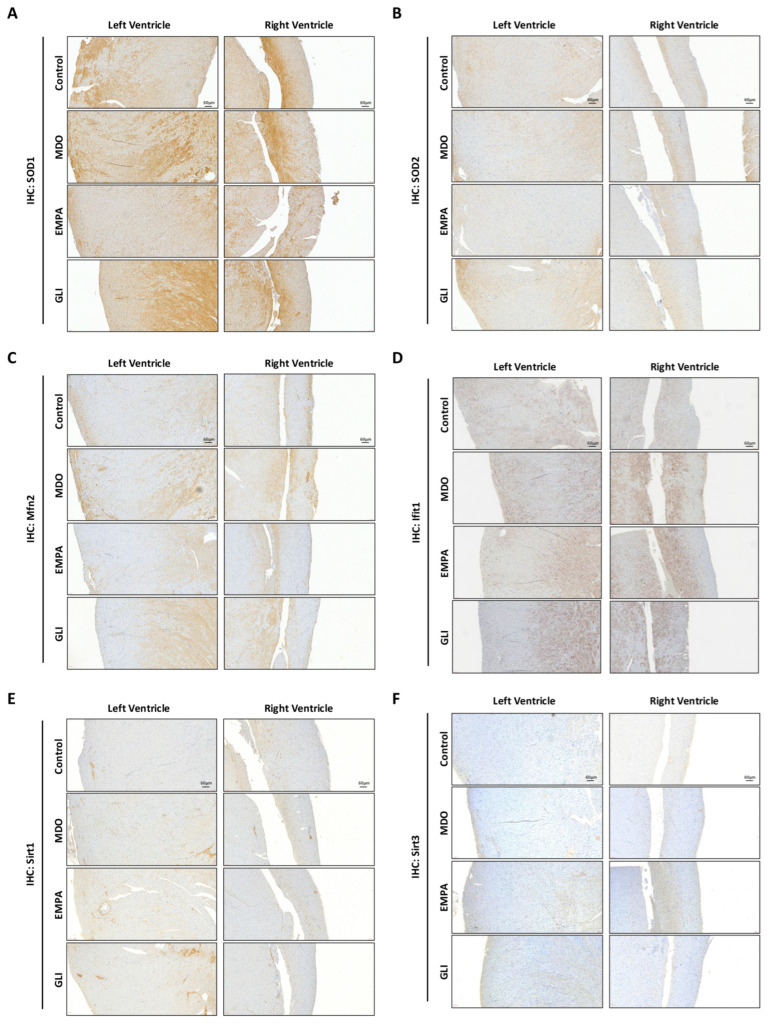
HFD-mouse model for verification of protein expression associated with mitochondrial regulation after treatment (*n* = 5 for each group). IHC histological of HFD mouse model for the analysis of (**A**) SOD1, (**B**) SOD2, (**C**) ifit-1, (**D**) mfn2, (**E**) Sirt1, and (**F**) Sirt3 expression regulated in individual mouse group. Left panel for left ventricle and right panel for right ventricle of cardiac tissue. Magnification: 200×.

Although we demonstrated the protective effects of the EMPA-related secretome in H9c2 cells, the corresponding biomarkers in mouse ventricular tissue needed to be evaluated for confirmation. Immunohistological analysis was used to evaluate the modifications of SOD1 and SOD2 in the ventricular tissues of mice. The results showed that the protein expression of SOD1 in the left ventricle was increased in the MDO group and was significantly reduced in the EMPA group compared with the control group. The expression in the GLI group was similar to that in the MDO group. In contrast, no significant change in SOD2 was found in either the MDO group or the EMPA group (Figure 5A,B). Regarding proteins that regulate mitochondrial homeostasis, the expression of ifit-1 and mfn2 was significantly increased in the MDO group but dramatically decreased in the EMPA group. However, there were no significant changes to the ifit-1 and mfn2 levels in the GLI group (Figure 5C,D). Furthermore, the modifying effects of the MDO and EMPA secretomes on the SIRT family proteins, SIRT1 and SIRT3, in myocardial ventricular tissue were determined. We found only a mild increase in SIRT1 expression in the MDO group. However, SIRT1 expression was significantly increased in the left ventricular tissue in the EMPA and GLI groups. Interestingly, SIRT3 expression was also slightly increased in the EMPA group, but not significantly (Figure 5E,F). This evidence suggests that the EMPA secretome might benefit myocardial ventricular tissue in part by enhancing mitochondrial function and reducing cell/mitochondrial OS.

## 3. Discussion

There were three main findings in our study. First, significant cardiotoxicity and ROS production in mitochondria were observed in the MDO group and were significantly attenuated in the EMPA group. Second, mitochondrial dysfunction caused by the epicardial secretome was significantly attenuated in the EMPA group compared with the MDO group. Third, the EMPA secretome modified the Ca^2+^ handling of mitochondria in cardiomyocytes. In the abovementioned alteration, the involved molecules’ regulation had also been investigated, and we found the expressions of target genes and proteins that may be dysregulated by MDO secretome induction and improved by EMPA treatment.

ROS products are unstable and can cause structural damage to DNA or proteins, which might cause reversible or irreversible damage to cells. Superoxide dismutase (SOD) can repair the damage caused by ROS but it still has limitations. Sometimes, the damage caused by ROS is irreversible and results in cellular dysfunction and viability. MDO might induce OS, which results in mitochondrial ROS accumulation and mitochondrial dysfunction. These effects can impact ATP production, cause mitochondrial Ca^2+^ overload, and impair mitochondrial biogenesis, possibly leading to cardiomyocyte dysfunction and cardiac arrhythmogenesis [17]. In cardiomyocytes, ROS accumulation increases the levels of inflammasomes and causes cardiac remodeling, which is the main pathological mechanism of heart failure [22,23,24]. In one animal study, insulin resistance was found to induce cardiac ROS in a manner independent of hyperglycemia, hyperlipidemia, and hyperinsulinemia [25]. In addition, ROS production is elevated in the myocardium in spontaneously hypertensive rats (SHR) [26]. In our study, ROS production was significantly lower after EMPA treatment than after MDO secretome treatment. ROS reduction was found both in whole cardiomyocytes and intra-mitochondria. ATP production in mitochondria was also enhanced by EMPA treatment. SGLT2is are next-generation antidiabetic medications. Beyond exerting glucose-lowering effects, they also provide additional cardiovascular benefits, effectively reducing the incidence of and hospitalization from heart failure in type 2 DM patients.

According to previous reports, the effects of MS on obese mice can be attenuated by reducing ROS and the associated OS as well as improving mitochondrial function [27]. Therefore, eliminating or reducing ROS in patients with MS might preserve cardiac function and attenuate the progression of heart failure. Given the cardiovascular effects of ROS, ROS production might be a promising marker to reflect the function of cardiomyocytes. Previous studies have revealed that glucose-lowering agents effectively eliminate ROS production and improve cardiovascular function [28,29]. In addition, previous studies have revealed that ROS can enhance the expression of inflammasomes and several cytokines, resulting in cardiac fibrosis [24]. The function of cardiac connexin is also impaired by ROS production [30]. In our previous study, cardiac fibrosis and connexin impairment were found to be attenuated after SGLT2i treatment [31].

ROS production and mitochondrial dysfunction in cardiomyocytes were attenuated in the EMPA group compared with the MDO group. In addition, several markers associated with mitophagy and mitochondrial biogenesis were significantly expressed in the EMPA group. Mitochondrial fusion, biogenesis, and mitophagy are associated not only with energy production but also with cardiovascular diseases, including heart failure [32]. The ifit family is associated with cellular interferon. In addition to the cellular immune response, they participate in apoptosis [33,34]. The increase in ifit-1 in the MDO group indicates the progression of apoptosis associated with OS, which has an impact on cardiomyocyte viability and could result in heart failure. In contrast, the secretome from the EMPA group might have reduced the cardiomyocyte apoptosis caused by the MDO stress response. In addition, the secretome from the EMPA group attenuated mitochondrial dysfunction in cardiomyocytes, as indicated by its effects on factors including SIRT1 expression and ATP production, compared with the dysfunction in the MDO group. SIRT1 is activated when mitochondria experience an energy deficiency or injury. SIRT1 expression increases sirtuin and PGC-1α expression, which might reduce OS and enhance mitochondrial biogenesis. This could preserve the quality and maintenance of mitochondria and improve the viability of cardiomyocytes. However, more evidence and investigation are needed to confirm this mechanism.

The homeostasis of Ca^2+^ in cardiomyocytes is important for preventing cardiac arrhythmia. Instability of Ca^2+^ can promote the extrusion of action potentials, which might cause early afterdepolarization (EAD) or delayed afterdepolarization (DAD), thus inducing arrhythmogenesis [35]. The association of mitochondria and cardiac arrhythmia has been examined and it has been found that the stabilization of mitochondria can reduce arrhythmogenesis [36]. The stability of Ca^2+^ is important not only in preventing arrhythmogenesis but also in cardiomyocyte contraction [37]. Our previous study showed that overload of the Ca^2+^ current in cardiomyocytes is decreased after SGLT2i treatment [6]. In the current experiment, mitochondrial Ca^2+^ overload was significantly increased in the MDO group but was relatively stabilized in the EMPA group. The results regarding JC-1 fluorescence in mitochondria were similar. JC-1 dye can aid in the discrimination of energized or deenergized mitochondria [38]. We found greater electro-stability not only in whole cardiomyocytes but also in mitochondria after EMPA secretome treatment. The expression of an associated protein, sarco-/endoplasmic reticulum Ca^2+^-ATPase (SERCA), was also increased in the MDO group and decreased in the EMPA group. OS, as reflected by ROS, triggers cellular calcium release and causes calcium overload in mitochondria [18]. An increase in SERCA expression occurs to maintain calcium homeostasis. The EMPA secretome decreased OS and maintained calcium homeostasis in cardiomyocytes.

These findings provide evidence that SGLT2i therapy could benefit cardiac arrhythmia and heart failure by reducing ROS levels and restoring mitochondria function. However, more experiments are needed to confirm our findings. However, there was no difference in NADH/NAD^+^ values between the two groups, implying that MDO and ROS production may damage mitochondria in the electron transport chain and eventually lead to dysfunction, such as decreased mitochondrial membrane potential and abnormal ATP production. More importantly, the secretome from the EMPA group attenuated this impairment of the dysfunction by restoring the membrane potential and ATP production of mitochondria in ventricle cardiomyocyte. This also means that the effect of the secretome of the MDO group on the activity of mitochondrial complex I-IV remains unknown, and the mechanism of how EMPA reverses these is also worthy of further investigation.

### Limitations

Our experiment had several limitations. First, the sample sizes in each group were limited. More study animals might be needed to confirm our results. Second, we found mitochondrial dysfunction in terms of ATP production. The effect on the electron transport chain of mitochondria was not clear. More studies on the expression of associated proteins might be needed. In addition, the change in mitochondrial biogenesis after EMPA secretome treatment may need to be examined in the future. Third, the exact mechanisms between the mitochondria and the sarcoplasmic reticulum require further investigation. The interaction of the mitochondria with the sarcoplasmic reticulum is important for calcium homeostasis, but the interactions of associated proteins, such as SERCA and RyRs, and mPTP, were not completely investigated in the current study. More studies are needed to elucidate the associations and interactions with regard to calcium metabolism.

## 4. Materials and Methods

We confirmed that all research methods were implemented in accordance with relevant guidelines and regulations, including an ethical statement stating that all research and article writing was completed by our laboratory.

### 4.1. Study Animal Preparation

A total of 20 C57BL/6J mice (age: 8 weeks) were divided into four groups (5 animals/group): (1) a control group, in which mice were fed standard chow for 16 weeks; (2) an MDO group, in which mice were fed a high-fat diet for 16 weeks; (3) an empagliflozin (EMPA) group, in which mice were fed a high-fat diet (Research Diets, Brunswick, NJ, USA) for 12 weeks and administered EMPA at 10 mg/kg daily for the following 4 weeks; and (4) a GLI group, in which mice were fed a high-fat diet for 12 weeks and administered GLI at 0.6 mg/kg daily for the following 4 weeks. After 16 weeks of feeding, these mice were sacrificed. Epicardial fat and heart tissue were collected for further investigation. The study protocol was approved by the Institutional Animal Care and Use Committee at Kaohsiung Medical University (KMU-107238).

### 4.2. Epicardial Fat Secretome Collection

The method of collecting the epicardial secretome was mainly based on our previous report [6]. C57BL/6J mice were intraperitoneally injected with pentobarbital (150 mg/kg) for anesthesia and then subjected to thoracotomy, and then the epicardial fat surrounding the epicardium of the heart was collected. After the adipose tissue was harvested, it was immersed in PBS and weighed. The adipose tissue was cut into small pieces and stored in a 12-well plate with serum-free DMEM added. The plate was then incubated in a CO_2_ incubator at 37 °C with gentle shaking. After approximately 18 h of incubation, the culture medium was collected and centrifuged at 4 °C for 10 min. The supernatant was collected and stored at −80 °C. The resulting secretome was quantified by a Bradford protein-binding assay. Experimental mice were purchased from Taiwan National Animal Center and the mice were euthanized at the designated time points using a high-flow rate of CO_2_. This study protocol was approved by the Institutional Animal Care and Use Committee at Kaohsiung Medical University and was reported in accordance to ARRIVE guidelines (https://law.moj.gov.tw/ENG/LawClass/LawAll.aspx?pcode=M0060027, last accessed on 28 March 2022).

### 4.3. Cell Culture

H9c2 was cultured according to our previous report. Briefly, H9c2 cells (ATCC CLR-1446; Rockville, MD, USA) were seeded onto collagen-coated dishes and cultured in DMEM supplemented with 10% fetal bovine serum (FBS). Culture conditions were 5% CO_2_ and 95% air at 37 °C. Cardiomyocytes at passage 20 to 40 were used [39].

### 4.4. Cell Viability Assay

3-(4,5-Dimethylthiazol-2-yl)-5-(3-carboxymethoxyphenol)-2-(4-sulfophenyl)-2H-tetrazole (MTS, Promega, Madison, WI, USA), for the colorimetric assay of cell viability, USA) was used to investigate changes in cell viability in accordance with previous reports. Briefly, 2.5 × 10^3^ cells were seeded in 96-well plates treated with separate mouse epicardial fat secretions from the control, MDO, EMPA, and GLI groups. Cells were treated with 80 μg/mL and 7.3 μg/mL of MTS and phenazine methyl sulfate (PMS, Sigma-Aldrich, St. Louis, MO, USA) for 4 h at the indicated time points. Absorbance was measured at 490 nm using a microplate reader (MTX Lab Systems, Inc., Vienna, VA, USA) and the percent of absorbance relative to cell viability was calculated.

### 4.5. Real-Time PCR Analysis

Total RNA was extracted using a NucleoSpin RNA Kit (Macherey-Nagel, Bethlehem PA, USA). Briefly, the cell lysate was applied to an RNA column and centrifuged. Subsequently, a membrane desalting buffer was added to the column and centrifuged. The RNase-free DNase reaction mixture was then added to each column to ablate the DNA. After digestion with DNase and centrifugation, the column containing RNA was transferred to a new tube and the RNA sample was purified by centrifugation. Finally, the samples were transferred to a new 1.5 mL tube and the RNA samples were dissolved in RNase-free H_2_O and stored at −80 °C. Complementary DNA (cDNA) was synthesized from the RNA using a reverse transcription reagent (Applied Biosystems, Bedford, MA, USA). The prepared cDNA was used for a quantitative polymerase chain reaction (qPCR) in a 96-well thermal cycler with the SYBR Green system to analyze gene expression. The gene primers are listed in Table 1.

### 4.6. Western Blot Analysis

Protein expression in H9c2 cells was investigated by western blot analysis. The experimental method used was described in our previous report [31]. The proteins obtained from the treated H9c2 cells were quantified by Bradford assay (Thermo Fisher) and separated by 10% SDS–PAGE. The proteins on the gel were transferred to Immobilon PVDF membranes (Millipore Corp) and the membranes were blocked for 1 h at room temperature. Then, primary antibodies were added and allowed to bind overnight at 4 °C. Secondary antibodies were then added and allowed to bind for 1 h at room temperature. Finally, an enhanced chemiluminescence reagent (Millipore Corp) was added to the PVDF membranes, which were photographed with a UVP Biospectrum, which is a fully automatic luminescence biomedical imaging system. The images were analyzed with LabWorks 4.5 Image Acquisition and Analysis software (Ultra-Violet Products Ltd., Cambridge, Cambs, UK.).

### 4.7. Immunohistological Analysis

Mouse hearts were harvested and fixed with 10% formalin. The specimens were dehydrated in a series of increasing alcohols, cleared with xylene, and finally embedded in paraffin. The paraffin specimens were cut into 5 mm thick sections with a microtome, flattened in a warm water bath, and mounted on glass slides. The slides were placed on a 40 °C baking tray for adherence. Afterwards, the slides containing the sections were subjected to dewaxing, followed by hematoxylin and eosin staining. Protein antibody staining was also conducted to detect SOD1, SOD2, ifit-1, mfn2, sirt1, and sirt3. The relevant images were processed with microscopy and the imaging analysis software ImageJ.

### 4.8. RNA Sequencing

RNA library construction and sequencing used Tools Biotech (BIOTOOLS, Taipei, Taiwan) reagents and followed the instruction manual. A TruSeqTM RNA LT Sample Prep Kit (Illumina Inc., San Diego, CA, USA) was used for isolation of total RNA and library construction. mRNA was randomly fragmented in the fragmentation buffer and cDNA was synthesized using random hexamers and reverse transcriptase. After first-strand synthesis, a custom-made second-strand synthesis buffer (Illumina), dNTPs, RNase H, and E. coli polymerase I were added to synthesize the second strand. A dsDNA HS reagent (Life Technologies, Carlsbad, CA, USA) and an Agilent 2100 Bioanalyzer (Agilent Technologies, Santa Clara, CA, CA) were used for test sample purification, end repair, ligation to sequencing adapters, and quantification of RNA concentration. The RNA pool was sequenced for 300 cycles on an Illumina HiSeq 2500 instrument (Illumina Inc., San Diego, CA, USA). The sequencing data were processed using Illumina software. The sequencing data was uploaded to the public database, Big Sub (https://ngdc.cncb.ac.cn/gsub/submit/gsa/list, submission ID: subCRA014864, last accessed on 18 January 2023).

### 4.9. Bioinformatic Analysis and IPA/KEGG/STRING Analyses

The raw FASTQ reads were used to verify the determined sequence quality. Linker sequences were removed, low-quality ends were trimmed, and low-quality reads were filtered on the basis of Q30 scores using Trimmomatic software. Then, RNA sequences were generated using low-quality data with the TopHat2 software. For sequence data analysis, the high-quality read alignments were compared with the human reference genome (grch38.p7). Gene counts were calculated by featureCounts software and the expression was normalized by the RLE/TMM/FPKM method. The differentially expressed genes (DEGs) were screened out with q value < 0.05 as the screening threshold. Upstream and downstream systems’ bioassays were performed on the DEGs using Ingenuity Pathway Analysis (IPA) and the Kyoto Encyclopedia of Genes and Genomes (KEGG) pathway database (http://www.genome.ad.jp/kegg/kegg1.Html, last accessed on 20 September 2022.

### 4.10. Mitochondrial Function Evaluation by JC-1 Assay

A total of 1 × 10^6^ H9c2 cells were washed twice with PBS and resuspended in 0.5 mL of JC-1 (Invitrogen, Molecular Probes; 5,5′,6,6′-tetrachloro-1,1′,3,3′-tetraethyl benzimidazolyl-carbocyanine iodide) working solution for 30 min at 37 °C in a 5% CO_2_ incubator. Next, the cells were centrifuged for 5 min (450× *g*; 5 min; room temperature), washed twice with PBS, and suspended in 0.5 mL of PBS. Thereafter, flow cytometry was used for fluorescence analysis. Healthy mitochondria were detected using green JC-1 monomers and red JC-1 aggregates; apoptotic or unhealthy mitochondria exhibited decreased levels of JC-1 dimers with red fluorescence.

### 4.11. Mitochondrial Adenosine Triphosphate (ATP) Production

H9c2 cells were co-cultured with epicardial fat in 24-well plates, harvested, resuspended in 100 µL of Tris-HCl (pH 7.5) containing 0.1 g/L digitonin (Calbiochem), and incubated on ice for 10 min. The cells were harvested and centrifuged at 750× *g* for 3 min at 4 °C and resuspended in 100 µL of Tris-HCl (pH 7.5). The oligomycin-sensitive ATP production rate was determined with a luminescence system. ATP production was monitored for 4 min after 5 min of incubation at 25 °C using the ATP monitoring reagent (BioThema AB, Handen, Sweden).

### 4.12. Mitochondrial NADH/NAD+ Synthesis

NAD+ and NADH were measured in a 96-well format by enzymatic cycling assays immediately after treated cell lysates were extracted. Then, 5 μL of NAD+ standard diluted cell extract was mixed with 95 μL of cycling mix. Cycling reactions were performed at room temperature for 30 min, and the accumulation of resorufin was measured by fluorescence with excitation at 544 nm and emission at 590 nm.

### 4.13. ROS Investigation Assay

H9c2 cells were seeded in 4-well chamber slides and treated with epicardial fat from the individual groups. After the indicated time point, the cells were incubated with CellROX Green Reagent (5 µM) and MitoSOX (5 µM) in DMEM at 37 °C for 30 min to observe the production of intracellular ROS and the content of ROS in mitochondria, respectively. DAPI was then used as a counterstain. After the cells were washed with PBS, green fluorescence (488 nm) and orange fluorescence (596 nm) were analyzed under a fluorescence microscope.

### 4.14. Determination of Mitochondrial Ca^2+^ Distribution

To measure the mitochondria-free Ca^2+^ distribution and accumulation, the Ca^2+^-sensitive-fluorescent dye Rhod-2 (Thermo Fisher, Austin, TX, USA) was used. Rhod-2 accumulates in mitochondria because of its high cationic charge, producing the colorless and nonfluorescent membrane-permeable molecule dihydro-Rhod-2 AM ester. Rhod-2 AM ester was incubated with specific amounts of NaBH4 for 10 min. Rhod-2 AM ester is red. According to the manufacturer’s instructions, dihydro-Rhod-2 AM ester can permeate cell membranes for cleavage of the AM ester and is oxidized to the dye Rhod-2, which produces Ca^2+^-dependent fluorescence in the mitochondrial environment.

### 4.15. Statistical Analysis

Descriptive data are expressed as counts, categorical variables are expressed as percentages, and continuous variables are expressed as the mean ± standard error of mean (SEM). One-way analysis of variance (ANOVA) and the Kruskal–Wallis test were used for comparison between groups, and post-hoc tests were calculated by the Bonferroni multiple comparison test. A subsequent *p* < 0.05 was defined as significant. All analyses were performed using SPSS version 20 (SPSS Inc., Chicago, IL, USA).

## 5. Conclusions

Our study reveals the adverse effects of metabolic disorder- and obesity-induced epicardial fat impairing cardiomyocyte H9c2 function by disturbing the mitochondrial function, such as reducing membrane potential, ROS accumulation, Ca^2+^ overload, and associated regulator expression. However, most of the dysregulation was improved by the EMPA secretome from epicardial fat compared with that of MDO group. These results provided evidence of the benefits of SGLT2i therapy in cardiac arrhythmia and heart failure. However, more investigations are required to confirm our findings.

## Figures and Tables

**Table 1:** Specific primer sequencing used for mitochondrial function evaluation.

Gene ID	Forward	Reserve
***mfn1***	TCAGAGCCTATCTTTCAGCTCC	ACGGACGCCAATCCTGTTAC
***mfn2***	AGCAAAGCTGCTCAGGAATAAA	CCTCTCCACGCAGACTCC
***mx1***	CACACCGTGACGGATATGGT	TTTGGACTTGGCGGTTCTGT
***mx2***	TGAACGTGCAGCGAGCTT	GGCTTGTGGGCCTTAGACAT
***zbp1***	ACGATTTACCGCCCAGAAGA	TCCAGCTGTTGGGTCCATTC
***ifit1***	ACAGCAACCATGAGGTTCTTTA	CCACAAGACATCAGAGAGGCT
***ifit1b***	CTTCATAGCACCATGAGTGAAGA	CAGGAATTTCAGGGGCTTCAA
***oasl***	CTGAAGGTAGTCAAGGTGGGC	TTTGTGATGCTTGGCTGCCT
***isg15***	ACAGCCATGGGCTGGGA	CCTTCAGCTCTGACACCGAC
***RYR1***	TTCTTCCCTGTCGTGAGCTT	CATGGAGCATAGCCAGGTG
***RYR2***	TTTTCTGGTTCCCTCGACTG	TTCAGAGCTTCTGGGCTCTC
***MCU***	AATTAAAGCATTGCAGGTGGA	CCCTTCTTCCCTCAGATCCT
***MICU1***	GACCCGGTAGATGGGAGAAT	CTTCAGCTGTCTCTGCATGG
***MCUR1***	TGCAAACTGTGGATCCGTAA	TTGAAAGTGTGGTCCATCCA
***VDAC1***	TGGCTCCATTTACCAGAAGG	CGACCTGATACTTGGCTGCT
***VDAC2***	CTGGGGACTTCCAGCTACAC	CCTGATGTCCAAGCAAGGTT
***VDAC3***	TTGACACAGCCAAATCCAAA	CTCCAAACTCAGTGCCATCA
***Bax***	TGTTTGCTGATGGCAACTTC	GATCAGCTCGGGCACTTTAG
***Bcl2***	AAGCTGTCACAGAGGGGCTA	CTCTCAGGCTGGAAGGAGAA
***ANT1***	GGCGACTGTCTCACCAAGAT	TCCGAAGTAGGCAGCTCTGT
***ANT2***	CAGCTGGATGATTGCACAGT	ATCAGTTCCTTTGCGTCCAG
***ANT3***	CAGCTGGATGATTGCACAGT	TCAGTTCTTTTGCGTCCAGA
***CypD-PPID***	AGGACTAGGTGTGGCAAGGA	CCAGTCATCCCCTTCTTTCA
***CypD-PPIF***	GTCAAAGAGGGCATGGATGT	AGCTCAACTGGCCACAGTCT
***MnSOD(SOD2)***	TGGTGCCTCTGGGTTTTCTA	ATCGGACAGGCCCTACCTAC
***SOD1***	GAGACCTGGGCAATGTGACT	TCATGGACCACCATTGTACG

## Data Availability

Not applicable.
